# Safety and Efficacy of Thulium Fiber Laser Versus Pneumatic Lithotripsy in Ureteric Stones During Semirigid Ureteroscopy

**DOI:** 10.5152/tud.2025.25011

**Published:** 2025-06-04

**Authors:** Abhay Dinkar Mahajan, Saiswaroop Yamajala, Sumeet Abhay Mahajan

**Affiliations:** 1 Department of Urology, Sai Urology Hospital, Aurangabad, India; 2 Department of Anesthesia, Sai Urology Hospital, Aurangabad, India

**Keywords:** Lithotripsy, pneumatic, thulium fiber laser, ureteric stone, ureteroscopy

## Abstract

**Objective:**

During the treatment of ureteric stones by semirigid ureteroscopy, pneumatic, and laser lithotripsy are commonly used for stone lithotripsy. This is the first prospective study to compare pneumatic with thulium fiber laser (TFL) lithotripsy for ureteric stones during semirigid ureteroscopy.

**Methods:**

A prospective evaluation was conducted on 100 patients, divided into group A (50 patients) who underwent TFL lithotripsy and group B (50 patients) who underwent pneumatic lithotripsy for ureteric stones treated by ureteroscopy. Urine culture and plain computed tomography (CT) scan were done in all the patients. Intraoperative stone clearance was assessed by endoscopic inspection and fluoroscopic evaluation. Postoperative stone clearance was evaluated at 7 days and 3 months by sonography and plain x-ray. Those patients with persistent or increased hydroureteronephrosis were further evaluated by CT scan to detect residual fragments and/or ureteric strictures.

**Results:**

The stone size, volume, and HU were comparable in both the groups. The lithotripsy time with TFL was significantly longer compared to pneumatic (12.41 vs. 5.16 minutes). Intraoperatively, the vision was better with TFL as compared to the pneumatic group (2 vs. 10 patients). Retropulsion was significantly less with TFL compared to pneumatic lithotripsy (2 vs. 10 patients). The complications and the stone-free rates were comparable in both the groups.

**Conclusion:**

Thulium fiber laser has distinct advantage of better vision and less retropulsion compared to pneumatic lithotripsy. It is also a safer modality as compared to the conventional pneumatic lithotripsy during the treatment of ureteric stones with ureteroscopy.

**Main Points**

- Thulium fiber laser (TFL) has significantly less retropulsion compared to pneumatic lithotripsy during the treatment of ureteric stones.
- The intraoperative visibility is better with the TFL during stone lithotripsy.
- Intraoperative complications and stone-free rates are comparable.
- Thulium fiber laser is a safe modality for stone lithotripsy in the ureter.

## Introduction

Ureteroscopy is a common procedure performed for ureteric stones. The European Urology Association recommends ureteroscopy as a single procedure with better stone-free rates as compared to shock-wave lithotripsy.^1^ Pneumatic lithotripsy is the most common method used for many years for stone lithotripsy. It mechanically breaks the stone with a “hammer-like effect,” and then the stone fragments are extracted with the help of forceps. Pneumatic lithotripsy is safe and effective, except the for chances of retropulsion of the stone in the kidney.^2^ Holmium:YAG (Ho:YAG) laser is considered as the gold standard for ureteric stone lithotripsy due to its effectiveness and ability to disintegrate all types of stone.^3^,^4^ However, Ho:YAG has some limitations like high cost, size, low energy, and difficulty in focusing the laser beam in small fibers.^5^ These limitations are overcome by the thulium fiber laser (TFL) as it has the capacity to generate low pulse energy, high frequency, can deliver an effective laser beam with smaller fibers, and has 4 times the stone ablation rate compared to the Ho:YAG laser.^6^,^7^ Many authors have compared Ho:YAG lithotripsy with pneumatic lithotripsy during ureteric stone treatment.^8^^-^^10^ This is the first study in the literature to compare conventional pneumatic lithotripsy with TFL lithotripsy for the treatment of ureteric stone during semirigid ureteroscopy.

## Material and Methods

### Study Design

A prospective randomized trial was conducted from April 2023 to May 2024 to compare the safety and efficacy of TFL with pneumatic lithotripsy during the treatment of ureteric stones using semirigid ureteroscopy. The study was approved by the Clinical Trial Registry of the country (CTRI number: CTRI/2023/04/051704, dated April 18, 2023) and by the Ethics Committee of Sai Urology Hospital bearing protocol number: ECRHS/SUH/01/2023, dated March 1, 2023. Informed consent was obtained from all the patients prior to the procedure. A sample size of 100 patients was calculated and was divided into 50 patients each in 2 groups. Group A consisted of patients who underwent thulium fiber lithotripsy, and group B consisted of patients underwent pneumatic lithotripsy during the surgery.

### Eligibility Criteria

All patients having ureteric stones at any location in the ureter were included in the study. Those patients who had both ureteric and renal stones and underwent simultaneous treatment for renal stones were excluded from the study.

### Study Procedure

All patients were randomly assigned in a 1 : 1 ratio to receive either pneumatic or laser lithotripsy. Randomization was done using an online randomizer tool (https://www.randomizer.org/). Urine culture, sonography, serum creatinine, and non-contrast computed tomography (CT) scan was done for all the patients. All patients underwent semirigid ureteroscopy (6.5 F Karl Storz, Germany) under spinal anesthesia. After placement of a Terumo glide wire under fluoroscopy guidance, a semi-rigid ureteroscope was passed into the ureter. Laser lithotripsy was done using a TFL 60-watt machine (Urolase SP, IPG Photonics, Moscow, Russia), with a 400-micron fiber. The laser settings during the fragmentation mode were an energy of 1 Joule and 6, 8, or 10 Hertz frequency, as preferred by the surgeon, depending on the stone’s hardness. In dusting mode, TFL was used with a setting of 0.1 Joule and 60 Hertz with a total power of 6 watts. A pneumatic lithotripter (Status, Satara, India) was used with a 1.2 mm rigid probe at a pressure of 2.5-3 kg/m^2^ with a frequency of 4 pulses/s. Fragments were extracted with forceps. During the procedure, the intra-operative vision was recorded by the surgeon according to the Likert score. The vision was categorized by the Likert scale into 3 categories as follows: grade 0—good vision, grade 1—average vision, and grade 2—poor vision requiring stoppage of lithotripsy till the vision gets cleared. Stone clearance was confirmed by fluoroscopy and endoscopically, and a double-J stent was placed in all the patients. Sonography and XR KUB were performed at 7 days and 3 months to assess the residual fragments. The double-J stents were removed between 10 and 14 days. Intraoperative complications like bleeding, poor vision, mucosal injuries, and stone migration were noted. Postoperative complications like fever, hematuria, sepsis, and residual stones were assessed. If the follow-up sonography showed persistent or increased hydroureteronephrosis, then the patients were further evaluated by CT scan to rule out ureteric stricture and/or residual stones.

### End Points

Primary end point was to assess the stone-free rate. The secondary end points were to evaluate intraoperative and postoperative complications.

### Statistical Analysis

The statistical analysis was conducted using SPSS Statistics software version 20.0 (IBM SPSS Corp.; Armonk, NY, USA). Descriptive analyses were performed for continuous variables, summarized as the number of observations, mean with standard deviation, or median with range. Categorical variables were presented as frequencies and percentages.

For inferential statistics, demographic data such as age were compared between groups using the Student’s *t*-test, while gender distribution was analyzed using the Chi-square test or Fisher’s exact test, as appropriate. Statistical tests were applied to various parameters, including demographic distribution, stone characteristics (location, size, number, and volume), energy rates for laser and pneumatic settings, intraoperative vision, intraoperative bleeding, retropulsion, and postoperative complications such as fever, pain, sepsis, hematuria, stone-free rates, and long-term complications.

All *P*-values were based on 2-sided significance tests, with a threshold for statistical significance set at *P* < .05.

## Results

The patients subjected to ureteric stone lithotripsy were divided into 2 groups—group A were those under TFL lithotripsy and group B pneumatic lithotripsy. Each group contained 50 patients. The demographics for age, sex, co-morbidities, and serum creatinine were equivalent in both the groups. The mean stone size was 9.64 mm in the laser group and 10.02 mm in the pneumatic group. The stone volume was 212 mm^3^ in the laser group and 288 mm^3^ in the pneumatic group. The mean HU was 938 and 770 in the laser and pneumatic group respectively. The difference between the stone laterization and the number of stones was not significant in both the groups (Table 1). The surgical time was comparable in both the groups, 68 minutes in the laser group, and 67 minutes in the pneumatic group. The pneumatic lithotripsy time of 5.16 minutes was significantly less compared to 12.41 minutes in the laser group (*P* = .0001). The mean laser energy was 1993 millijoules, the stone ablation time was 0.40 mm^3^/s, and the laser ablation efficiency was 12.14 Joules/mm^3^. As the pneumatic machine does not have energy value, this parameter was evaluated only in the laser group. In most of the patients, the TFL was used in fragmentation mode (39 patients) and dusting mode was used in a smaller number of patients (11 patients). The most preferred laser setting in the fragmentation mode was 1 J and 6 Hz with a total of 6 watts (Table 2). Similarly, when used in dusting mode, the maximum power never exceeded 6 watts (0.6 J X 60 Hz). Intra operative bleeding was observed in 3 patients in the laser group and only 1 patient in the pneumatic group; however, the vision was poor in only 2 patients in the laser group vs. 10 patients in the pneumatic group. The difference was statistically significant (*P* = .031). There were no significant intraoperative complications in both the groups. Retropulsion was significantly higher in the pneumatic group (10 patients) compared to the patients undergoing laser lithotripsy (2 patients) (*P* = .031) (Table 3). There was no significant difference in postoperative hematuria and fever in both the groups. Two patients in the laser lithotripsy group had Clavien-Dindo grade I complications, and in the pneumatic group, 5 and 1 patient had Clavien-Dindo I and II complications, respectively. The intraoperative complications were also graded according to the modified Satava classification for ureteroscopy complications. Grade I complications included minor mucosal injury and bleeding observed in 5 and 11 patients in the laser and pneumatic groups, respectively. Two patients in the pneumatic group required flexible ureteroscopy in the same sitting for retropulsion of the stone and hence were included in grade IIa complications. No ureteric stricture was encountered in the TFL lithotripsy group. Stone-free rates were assessed by sonography and x-ray KUB at 7 days and 3 months. The stone-free rates were 94% by laser lithotripsy and 92% by pneumatic lithotripsy (Table 4). All the fragments passed off from the ureter at 3 months, resulting in 100% stone-free rates in both the groups.

## Discussion

Treatment of ureteric stones at all locations by semirigid ureteroscopy is a very common procedure performed by urologists. Pneumatic lithotripsy is commonly used for stone lithotripsy, which works by compressed air producing a ballistic effect at the tip of the probe, leading to stone fragments.^11^ A higher rate of stone retropulsion with pneumatic lithotripsy has led to a gradual shift toward laser lithotripsy as the preferred treatment method.^12^ Holmium:YAG laser has been widely used for stone lithotripsy, for renal as well as ureteric stones, due to its effectivity and potential to treat all types of stone composition.^4^ Subsequently, there were many studies which compared pneumatic and Ho:YAG laser for the treatment of ureteral stones.^13^,^14^ Since the introduction of TFL in 2017, it has been preferred for stone lithotripsy due to its distinct advantage over Ho:YAG in terms of its capability to generate low energy and high frequency, with effective laser delivery through smaller fibers and having 4 times the ablation rate over Ho:YAG laser.^6^,^7^ Pre-clinical study by Andreeva et al^15^ showed that use of TFL resulted in excellent dusting and reduced retropulsion during laser lithotripsy. The effectivity of TFL was further evaluated for ureteric stones^16^ and was also compared with the Ho:YAG laser during ureteric lithotripsy.^7^ This is the first study which compared the safety and efficacy of TFL vs. the conventional pneumatic lithotripsy.

In this study, the lithotripsy time with TFL was significantly longer compared to the pneumatic group. This is attributed to the low-energy settings of 6 watts in most of the patients. Similar findings were observed in the Ho:YAG studies using low energy settings.^17^,^18^ When TFL was compared with Ho:YAG, TFL had significantly shorter operative time and laser time. Despite the longer laser time, the total operative time did not show any significant difference between the 2 groups (68.8 minutes vs. 67.31 minutes). This was probably because, although the pneumatic lithotripsy was faster, the extraction of fragments with the forceps took more time, while the laser produced more dust and less fragments to be extracted at the end of the stone lithotripsy.

Significantly less retropulsion was observed with TFL as compared to pneumatic lithotripsy (*P* = .031). It is considered an important parameter during the treatment of ureteral stones, as stone migration in the kidney requires additional procedure like flexible ureteroscopy or staging of the procedure. Retropulsion also adds to increased surgery time and additional cost of the surgery. Similar findings were observed by Enikeev et al^16^ in the treatment of ureteral stones by TFL, where there were no patients with retropulsion of the stone in the kidney.

Intraoperatively, it was observed that the visibility was much better with TFL as compared to pneumatic lithotripsy (*P* = .031). Similar findings were stated in a systemic review by Traxer et al,^19^ and the visibility was also maintained during higher TFL frequency settings.^20^ This is attributed to the smaller laser fiber size compared to the pneumatic probes, which leads to better irrigation and good visibility with laser lithotripsy.

The immediate stone-free rate at 7 days was 94% with TFL and 92% with pneumatic lithotripsy, and at 3 months it was 100% after evaluation by sonography and XR KUB. Similar higher stone-free rates with TFL were observed by Martov et al,^7^ where there were no residual stones at one month of treatment with TFL. Like this study, Akdeniz et al^10^ also stated that the success of ureteroscopy is approaching 100%. In their series, they had an overall success rate of 89.9% with pneumatic and 87.9% with laser lithotripsy.

A very low complication rate was observed in both groups, with Clavien-Dindo grade I in the TFL and grade I and II in the pneumatic lithotripsy group. There were no major complications in either group.

In a meta-analysis of Ho:YAG laser and pneumatic lithotripsy by Chen et al^9^ a higher ureteric stricture rate was found in the laser lithotripsy group. In this study, no post-operative ureteric stricture was encountered with TFL lithotripsy. This is attributed to multiple factors like very low laser settings, good irrigation, and disimpacting the stone prior to the use of the laser. To elaborate further, most of the patients were treated with TFL using a maximum of 6 watts of total power. The majority of the patients were treated in fragmentation mode with high energy and low frequency. In dusting mode, due to high frequency, it is difficult to stabilize the laser fiber in the ureter and, in addition might generate high temperature, which might lead to mucosal damage. Enikeev et al^16^ suggested laser settings of 15 watts for ureteroscopic (URS) lithotripsy in different combinations. It is suggested that no more than 6 watts of power be used with TFL during URS lithotripsy for ureteric stones.

A unique approach to laser lithotripsy was employed, which includes working with the laser fiber at the center of the stone, safeguarding the ureteral mucosa, and using the laser beam in an interrupted fashion. All these factors and precautions probably resulted in no ureteric strictures during the study.

The strength of the study is that it is a prospective randomized study, and all the surgeries were performed by a single surgeon. This is the first study, to our knowledge, that has compared the conventional pneumatic lithotripter with the TFL during ureteric stone lithotripsy.

The only limitation in the study was that not all patients were evaluated by CT scan during the follow-up. It is assumed that during semirigid ureteroscopy, it is reliable to assess the stone-free rate by fluoroscopy and endoscopic inspection of the complete ureter after the end of the procedure. Also, the worrisome complication of ureteric stricture post-laser or pneumatic lithotripsy will subsequently lead to hydroureteronephrosis. Hence, only patients having persistent or increased hydronephrosis were subjected to a CT scan during the follow-up.

Thulium fiber laser has distinct advantage of providing good vision and significantly less retropulsion compared to pneumatic lithotripsy. The stone-free rates and complication rates were similar when compared to the conventional pneumatic lithotripsy for ureteric stones using semi-rigid ureteroscopy. Thulium fiber laser lithotripsy is a safe modality for the treatment of ureteric stones.

## Figures and Tables

**Table 1.** Demography, Comorbidities, and Stone Characteristics, and Stone Location in Patients Undergoing Laser and Pneumatic Lithotripsy

Parameter		Laser	Laser	Pneumatic	Pneumatic	*P*
Gender		No. of Patients	%	No. of Patients	%	
Male		40	80	34	68	-
Female		10	20	16	32	-
**n**		50	100	50	100	-
Mean Age		41.56	-	41.66	-	-
SD (Age)		15.8	-	15.95	-	-
**Comorbidities**						
HTN		4	36.36	8	50	-
Psychosis		1	9.09	-	-	-
DM + HTN		4	36.36	5	31.25	-
Hypothyroidism		1	9.09	-	-	-
DM		-	-	1	6.25	-
IHD		-	-	1	6.25	-
SLE		-	-	1	6.25	-
Mean S.Creat		1.52	-	1.41	-	-
SD		1.73	-	1.55	-	-
**Stone Size and Density**						
		**Laser Lithotripsy**		**Pneumatic Lithotripsy**		
	**N**	**Mean**	**SD**	**Mean**	**SD**	
Largest dimension of stone (mm)	50	9.64	2.68	10.02	3.55	.8976
NCCT volume (cubic mm)	50	212.77	239.96	288.22	343.37	.2058
NCCT HU of stone	50	938.72	452.29	770.63	490.84	.1964
**Location of Ureteric Stones**						
		**No. of Patients**	**%**	**No. of Patients**	**%**	.173
Upper		17	34	12	24	
Mid		6	12	4	8	
Lower		25	50	30	60	
Multiple stones		2	4	4	8	
**Side (LT = Left, RT = Right, B/L = Bilateral)**						
		**No. of patients**	**%**	**No. of patients**	**%**	.705
Bilateral (B/L)		1	2	2	4	
Left (LT)		22	44	25	50	
Right (RT)		26	52	23	46	

**Table 2.** Surgical and Lithotripsy Parameters in Patients Undergoing Laser and Pneumatic Lithotripsy

Parameter	Laser Lithotripsy		Pneumatic Lithotripsy		*P*
	Mean	SD	Mean	SD	
Surgery time (minutes)	68.82	17.62	67.31	20.17	.691
Lithotripsy time (minutes)	12.41	511.27	5.16	223.16	**.0001**
Stone ablation time (mm^3^/s)	0.40	0.51	NA	NA	NA

**Surgery Details**	**Group**	**Mean**		**SD**	
Laser energy (millijoules)	Laser	1993.40		1983.70	
Laser stone ablation efficacy (J/mm^3^)		12.14		12.35	
**Laser Lithotripsy**					
Fragmentation mode	**Energy (J)**	**Frequency (H)**	**Total Power (W)**	**Number of Patients N**	**%**
	1	6	6	32	91.43
	1	8	8	1	2.86
	1	10	10	6	17.14
Dusting mode	0.1	60	6	11	31.43

**Table 3.** Intraoperative Safety Parameters in Patients Undergoing Laser and Pneumatic Lithotripsy

Safety Parameters	Laser Lithotripsy		Pneumatic Lithotripsy		Total No. of Patients	*P*
	No. of Patients	%	No. of Patients	%		
Retropulsion	2	4.0	10	20.0	50	**.031**
Intraoperative bleeding	3	6.0	1	2.0	50	0.61
Intraoperative poor vision	2	4.0	10	20.0	50	**.031**
Intraoperative complication	0	0.0	0	0.0	50	NA

**Table 4.** Postoperative Safety Parameters and Stone-Free Rates in Patients Undergoing Laser and Pneumatic Lithotripsy

Safety Parameters	Laser Lithotripsy		Pneumatic Lithotripsy		Total No. of Patients	*P*
	No. of Patients	%	No. of Patients	%		
Post-op Hematuria	2	4.0	1	2.0	50	1.000
Fever	2	4.0	5	10.0	50	.433
Sepsis	0	0.0	0	0.0	50	NA
Clavien-Dindo classification I	2	4.0	5	10.0	50	.269
Clavien-Dindo classification II	0	0.0	1	2.0	50	.269
Modified Satava classification 1	5	10.0	11	22.0	50	.267
Modified Satava classification 2a	0	0.0	2	4.0	50	.495
USG at 7 Days (residual fragment)	4	8.0	3	6.0	50	1.000
X-ray at 7 Days (residual fragment)	2	4.0	3	6.0	50	1.000
Hydronephrosis (HN)	0	0.0	0	0.0	50	NA
USG/KUB Residual Fragment at 3 Months	0	0.0	0	0.0	50	NA
Stone-Free Status at 7 Days	47	94.0	46	92.0	50	1.000
Stone-Free Status at 3 Months	50	100.0	50	100.0	50	NA

## Data Availability

The data that support the findings of this study are available on request from the corresponding author.
